# Flux analysis of the *Lactobacillus reuteri* propanediol-utilization pathway for production of 3-hydroxypropionaldehyde, 3-hydroxypropionic acid and 1,3-propanediol from glycerol

**DOI:** 10.1186/1475-2859-13-76

**Published:** 2014-05-27

**Authors:** Tarek Dishisha, Luciana P Pereyra, Sang-Hyun Pyo, Robert A Britton, Rajni Hatti-Kaul

**Affiliations:** 1 Department of Biotechnology, Center for Chemistry and Chemical Engineering, Lund University, SE-221 00 Lund, Sweden; 2 Department of Microbiology and Molecular Genetics, Michigan State University, East Lansing, Michigan, USA

**Keywords:** *Lactobacillus reuteri*, 3-hydroxypropionaldehyde, 3-hydroxypropionic acid, 1,3-propanediol, Biodiesel glycerol, Flux analysis, Biorefinery, Biochemicals

## Abstract

**Background:**

*Lactobacillus reuteri* converts glycerol to 3-hydroxypropionic acid (3HP) and 1,3-propanediol (1,3PDO) via 3-hydroxypropionaldehyde (3HPA) as an intermediate using enzymes encoded in its propanediol-utilization (*pdu*) operon. Since 3HP, 1,3PDO and 3HPA are important building blocks for the bio-based chemical industry, *L. reuteri* can be an attractive candidate for their production. However, little is known about the kinetics of glycerol utilization in the Pdu pathway in *L. reuteri*. In this study, the metabolic fluxes through the Pdu pathway were determined as a first step towards optimizing the production of 3HPA, and co-production of 3HP and 1,3PDO from glycerol. Resting cells of wild-type (DSM 20016) and recombinant (RPRB3007, with overexpressed *pdu* operon) strains were used as biocatalysts.

**Results:**

The conversion rate of glycerol to 3HPA by the resting cells of *L. reuteri* was evaluated by *in situ* complexation of the aldehyde with carbohydrazide to avoid the aldehyde-mediated inactivation of glycerol dehydratase. Under operational conditions, the specific 3HPA production rate of the RPRB3007 strain was 1.9 times higher than that of the wild-type strain (1718.2 versus 889.0 mg/g_CDW_.h, respectively). Flux analysis of glycerol conversion to 1,3PDO and 3HP in the cells using multi-step variable-volume fed-batch operation showed that the maximum specific production rates of 3HP and 1,3PDO were 110.8 and 93.7 mg/g_CDW_.h, respectively, for the wild-type strain, and 179.2 and 151.4 mg/g_CDW_.h, respectively, for the RPRB3007 strain. The cumulative molar yield of the two compounds was ~1 mol/mol glycerol and their molar ratio was ~1 mol_3HP_/mol_1,3PDO_. A balance of redox equivalents between the glycerol oxidative and reductive pathway branches led to equimolar amounts of the two products.

**Conclusions:**

Metabolic flux analysis was a useful approach for finding conditions for maximal conversion of glycerol to 3HPA, 3HP and 1,3PDO. Improved specific production rates were obtained with resting cells of the engineered RPRB3007 strain, highlighting the potential of metabolic engineering to render an industrially sound strain. This is the first report on the production of 3HP and 1,3PDO as sole products using the wild-type or mutant *L. reuteri* strains, and has laid ground for further work on improving the productivity of the biotransformation process using resting cells.

## Background

Recent years have seen a growing interest in shifting from fossil- to a more renewable feedstock based on biomass for the production of chemicals and materials with a lower carbon footprint [1,2]. In order to match the efficiency and flexibility of the petrochemical industry, a number of platform chemicals have been identified for the bio-based industry that would serve as building blocks for a range of other products [1,2]. Among these are 3-hydroxypropionaldehyde (3HPA), 3-hydroxypropionic acid (3HP) and 1,3-propanediol (1,3PDO) [1,2]. While there is no existing industrial production of the former two chemicals [3,4], 1,3PDO has been produced industrially from fossil-based propylene and ethylene, respectively, by Degussa and Shell processes (in both processes, 3HPA is formed as an intermediate) [5,6].

3HPA is a potential platform for several high-volume products like acrolein, 3HP, 1,3PDO, malonic acid, acrylamide and acrylic acid [3,7-9], and can also be used as an antimicrobial agent (reuterin) in food and health industries [3]. 3HP, besides being an important precursor for acrylic acid [10], is a potential building block for the production of propionilactone, biodegradable polyesters and oligomers, and other products for food and cosmetic industries [10-12]. 1,3PDO is incorporated in copolyesters and advanced polymers, and used as ingredient in wood paints, anti-freeze, adhesives and laminates [10,13,14]. Microbial production offers an attractive route for obtaining these chemicals from bio-based resources, as seen by the several studies reported using wild-type and engineered bacteria. Production of 1,3PDO from sugar using engineered *Escherichia coli* is indeed done on large scale [15,16], and scale up of 3HP production is also being attempted [17].

The large volume of glycerol obtained as a by-product of biodiesel as well as bioethanol and soap manufacture [18-21], represents a potentially useful carbon substrate for production of 3HPA, 3HP and 1,3PDO. Several members of the genera *Clostridia, Lactobacilli, Klebsiella*, and *Citrobacter* can use glycerol as an electron acceptor yielding 1,3PDO via 3HPA as an intermediate. Recently, the feasibility of simultaneous production of 3HP and 1,3PDO using recombinant strains of *Klebsiella pneumonia, Lactobacillus reuteri* and *E. coli* has been reported [4,22-26]. The simple separation of these two compounds makes this route very attractive [27]. However, despite the high titers and production rates reported, most of these production routes share the common problem of relatively low yields and large amounts of by-products (lactic acid, ethanol, butanol, succinic acid, and acetic acid, among others) with high structural similarity to the desired products, which complicated the downstream processing [4,23,24,26]. Further metabolic engineering of *K. pneumonia* aimed at minimizing lactic acid production was successful, but the cumulative yield of 3HP and 1,3PDO was only 0.77 mol/mol glycerol [26].

*L. reuteri* is a very attractive candidate for the production of 3HPA, 3HP and 1,3PDO. In contrast to the opportunistic pathogen *K. pneumonia*, it has a "generally recognized as safe" status and is also used as a probiotic, hence restrictions on scaling up for industrial production are minimal. *L. reuteri* uses glycerol only as an electron acceptor and not as a carbon source for growth, which ensures the absence of undesired by-products in the reaction mixture. Transformation of glycerol by growing *L. reuteri* results in 1,3PDO as the main product. The resting cells, on the other hand, convert glycerol to 3HPA catalyzed by glycerol dehydratase (GDH), while 1,3PDO and 3HP are formed in small quantities as by-products. Due to the inhibitory effect of 3HPA, the process is rapidly terminated if the aldehyde is not trapped as a complex with sodium bisulfite, semicarbazide or carbohydrazide [28-31]*.* The ability of *L. reuteri* to synthesize adenosylcobalamin (vitamin B12), an essential co-factor for glycerol dehydratase, is an additional economical advantage for its use as production host [32].

Bioconversion of 3HPA to 1,3PDO and 3HP occurs through a reductive and an oxidative pathway, respectively [28,29]; the enzymes involved in these reactions are encoded in the propanediol-utilization (*pdu*) operon. The production of 3HP alone or in a mixture with 1,3PDO as main products using a wild-type *L. reuteri* has however not been successful [22,33], mainly due to the accumulation to toxic levels of the intermediate 3HPA [22,28] and its inhibitory effect on one of the enzymes of the oxidative pathway [34], as well as the diversion of glycerol to dihydroxyacetone (DHA) catalyzed by a glycerol dehydrogenase [33]. The co-production of 3HP and 1,3PDO was only possible after the gene encoding the enzyme glycerol dehydrogenase was knocked out [22]. Despite the increasing interest and research on the utilization of *L. reuteri* in industrial biotechnology, there is only fragmentary information regarding metabolic fluxes within the Pdu pathway and conditions for the co-production of 3HP and 1,3PDO as main end products.

In the present study, metabolic flux analysis (MFA), a technique that has been widely used for quantification of fluxes, determination of nodal rigidity and metabolic bottlenecks, and assisting the choice of the proper metabolic engineering strategy [35-37], was employed to gain a better understanding of the kinetics of glycerol utilization and 3HPA distribution into reductive and oxidative pathways in *L. reuteri*. This was done with the aim to determine conditions for achieving maximal yields of the desired products while overcoming the inhibitory/toxic effects. The determination of these fluxes is challenging due to the toxic nature of the intermediate 3HPA, the compartmentalization of some intermediates (3HPA and 3HP-CoA) [38], and the co-factors recycling between the different steps, affecting the overall dynamics of the system. The flux of glycerol to 3HPA was analyzed using batch mode of operation by trapping the aldehyde as a complex, while the fluxes to 3HP and 1,3PDO were measured using a multi-step fed-batch mode of operation. The study was performed with two *L. reuteri* strains: wild-type and an engineered strain (RPRB3007) [39] in which the genes encoded in the *pdu* operon were overexpressed.

## Results and discussion

### Mechanism of glycerol biotransformation and choice of biotransformation conditions with whole cells of *L. reuteri*

**Figure 1:**
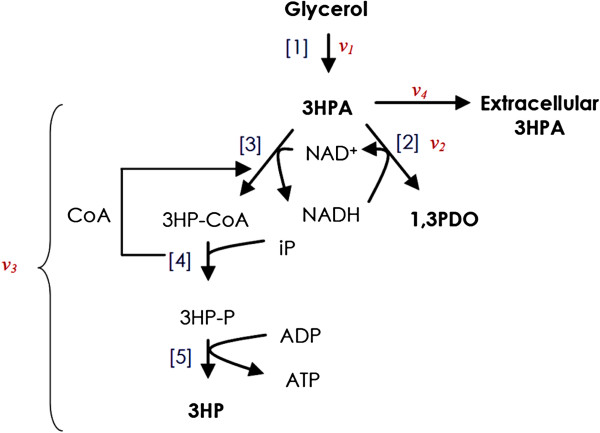
**Metabolic pathway for transformation of glycerol in *L. reuteri.*** Proposed metabolic pathway for biotransformation of glycerol to 3HP and 1,3PDO via 3HPA as intermediate by resting cells of *Lactobacillus reuteri*. The different enzymes and co-factors involved are shown. Enzymes: [1] B12-depdendent glycerol/diol dehydratase (PduCDE), [2] 1,3-propanediol oxidoreductase (PduQ), [3] propionaldehyde dehydrogenase (PduP), [4] phosphotransacylase (PduL), and [5] propionate kinase (PduW). ν_*i*_ represent the specific reaction rate.

The expected pathway for the conversion of glycerol to 1,3PDO and 3HP by *L. reuteri* is illustrated in Figure 1. It is initiated by the dehydration of glycerol to 3HPA in a reaction catalyzed by a B12-dependent glycerol/diol dehydratase (GDH) [33]. Further conversion of 3HPA to 1,3PDO is catalyzed by 1,3-propanediol oxidoreductase (PduQ) and a putative alcohol dehydrogenase [40]. This reaction is coupled to the regeneration of one mole of NAD^+^ per mole 1,3PDO produced, allowing the glycerol to be continuously utilized by the growing cells as an electron acceptor for conversion of NADH generated during sugar metabolism and cell growth [41]. In contrast, when resting cells are used, this reaction will proceed only until all the NADH molecules available in the cells are consumed. At this point, the role of the oxidative pathway is very important through which 3HPA is oxidized in a three-step reaction catalyzed by CoA-dependent propionaldehyde dehydrogenase (PduP), phosphotransacylase (PduL) and propionate kinase (PduW), respectively, to 3HP with 3HP-CoA and 3HP-phosphate as intermediates, and utilizing NAD^+^ to yield NADH resulting in a balance of redox equivalents [33,42]. The overall balance of reducing equivalents would result in the equimolar production of the hydroxyacid and the diol. Furthermore, the Coenzyme A (CoA) required for synthesis of 3HP-CoA in the PduP-catalyzed step is released later in the PduL-catalyzed reaction allowing continuous regeneration of these two co-factors. The last step of the conversion of 3HPA to 3HP yields one mole of ATP per mole of 3HP formed, which makes the reaction thermodynamically favorable [27], and could be a reason for continuity even when resting cells are used. The ATP generated during this step might be used for maintenance of cells [22] or for active transport of 3HP outside the cell [43]. Also, it could be utilized for reactivation (via a reactivase) of the inactivated glycerol dehydratase in a process that requires ATP, Mg^+2^ ions and adenosylcobalamin [44].

Compared to other species that can grow on glycerol, *L. reuteri* cannot use glycerol as a carbon source due to the lack of dihydroxyacetone kinase for conversion of DHA to dihydroxyacetone phosphate prior to metabolism via the glycolysis and phosphoketolase pathways [45,46]. This implies that no by-products will be formed during glycerol bioconversion, hence simplifying the downstream processing that is suggested to be one of the main factors influencing the cost of production of 3HP by recombinant *E. coli*[47].

Based on the above information, the process using resting cells of *L. reuteri* was selected to analyze the metabolic flux of glycerol to 3HPA, and the flux distribution around 3HPA to the reductive and oxidative pathway branches. *L. reuteri* cells used for the studies were grown in the culture medium containing 5 g/L 1,2-propanediol (1,2PDO), which (or glycerol) is important for activating the expression of the genes encoding enzymes and structural proteins required for glycerol metabolism [48] and also for triggering the formation of metabolosomes required for entrapment of the produced aldehyde and its subsequent conversion to the CoA-derivative through the membrane-bound PduP [33]. Addition of 1,2PDO results in a larger increase in GDH activity than with the same concentration of glycerol [33].

Cell growth was continued until the late stationary phase (11 h) when all the glucose (40 g/L) and 1,2PDO were consumed, and yielding a final cell density of 3.1 g_CDW_/L, 14.3 ± 0.4 g/L lactic acid, 2.5 ± 0.3 g/L acetic acid and 6.6 ± 1.6 g/L ethanol. Also 3.0 ± 0.4 g/L *n*-propanol was obtained from 1,2PDO. The resulting active biomass was utilized as a whole-cell catalyst for biotransformation experiments.

### Metabolic flux of glycerol in *L. reuteri* resting cells using batch mode of operation in the presence of 3HPA scavenger

According to our earlier experiments, resuspension of the *L. reuteri* cells, cultivated as described above, in a glycerol solution (200 – 400 mM) was suitable for the production of 3HPA at a high purity and concentration, but loss of cell viability and enzymatic activity was observed within 2 h [28,49]. This could be attributed to different factors, including toxicity of the aldehyde to the cells and enzymatic machineries [49], and the inactivation of the glycerol dehydratase enzyme due to breakdown of the Co-C bond in the co-factor adenosylcobalamin [44].

**Figure 2:**
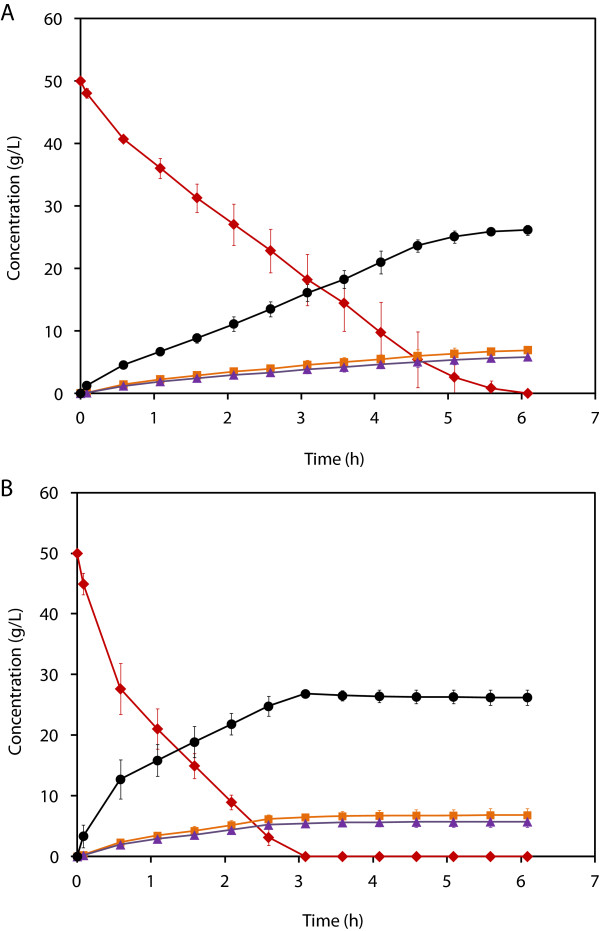
**Batch transformation of glycerol using resting cells of *L. reuteri *with *in situ *3HPA complexation.** Time course for the batch biotransformation of glycerol (50 g/L) using resting cells of *L. reuteri* (6 g_CDW_/L), wild-type **(A)** and RPRB3007 **(B)** in the presence of 50.6 g/L carbohydrazide. The concentrations of glycerol (◆), 1,3PDO (▲), 3HPA (**●**) and 3HP (■) are shown. Biotransformation (500 mL working volume) was performed in a 1-L bioreactor under anaerobic conditions at 37°C, 500 rpm and pH 7 (adjusted with 5 N NH_4_OH).

The biocatalyst life span is significantly extended by *in situ* complexation of the aldehyde with bisulfite, carbohydrazide or semicarbazide [28,29]; the latter two form more stable complexes with 3HPA [29]. Hence, carbohydrazide was chosen as a 3HPA scavenger to minimize the toxic effect of the aldehyde on the cells and hence allow determination of the true conversion rate of glycerol to 3HPA. pH 7 was selected for determining the glycerol uptake and 3HPA accumulation rates based on the earlier report revealing the optimal pH of production of 3HPA to be shifted from pH 5 to 7 in the presence of carbohydrazide [29]. Under operational conditions, the conversion of 50 g/L glycerol was achieved within 6 h using 6 g_CDW_/L of the wild-type strain (Figure 2A). The overall volumetric- (*Q*_*3HPA*_) and specific 3HPA production rates (*q*_*3HPA*_) were 4.3 ± 0.1 g/L.h and 889.0 ± 65.6 mg/g_CDW_.h, respectively. When RPRB3007 strain was used, the specific productivity was 1.9x higher (1718.2 ± 98.3 mg/g_CDW_.h) than in the wild-type strain and complete consumption of glycerol was achieved within 3 h with formation of 26.2 ± 1.3 g/L 3HPA at a rate of 8.7 g/L.h as a carbohydrazide complex (Figure 2B). Simultaneously, 5.8 g/L 1,3PDO and 6.9 g/L 3HP were also obtained. In both strains, glycerol uptake and 3HPA production was characterized by a fast rate for the first 30 min followed by a slower linear rate thereafter (Figure 2 and Table 1).

**Table 1:** **Specific rates for glycerol consumption and 3HPA, 3HP and 1,3PDO production by *L. reuteri***

**Mode**	**Glycerol feeding rate (g/h)**	**pH**	**Specific production/consumption rates (mg/g**_**CDW**_**.h)**							
			**Glycerol (*q***_***S***_**)**		**3HPA (*q***_***3HPA***_**)**^**[c]**^		**3HP (*q***_***3HP***_**)**		**1,3PDO (*q***_***1,3PDO***_**)**	
			***v***_***1***_		***v***_***4***_		***v***_***3***_		***v***_***2***_	
			**WT**	**M**	**WT**	**M**	**WT**	**M**	**WT**	**M**
**B**^**[a] **^**(overall)**	--	7	-1583.2 ± 204.8	-2842.0 ± 324.8	889.0 ± 65.6	1718.2 ± 98.3	233.8 ± 43.7	426.5 ± 40.5	197.5 ± 36.9	360.3 ± 34.2
**B**^**[b] **^**(linear)**	--		-1474.3 ± 182.8	-2101.3 ± 252.3	856.2 ± 79.3	1122.6 ± 108.8	200.5 ± 41.2	330.2 ± 36.1	169.3 ± 34.8	291.6 ± 48.4
**FB**	0.6	7	-108.0 ± 1.6	-98.7 ± 5.8	0	0	49.3 ± 3.5	49.8 ± 1.6	41.7 ± 3.0	42.1 ± 1.3
	1.6		-266.2 ± 15.7	-256.9 ± 3.2	29.0 ± 5.8	0	110.8 ± 3.0	113.5 ± 2.3	93.7 ± 2.5	95.9 ± 2.0
	2.5		ND	-420.8 ± 5.8	ND	36.2 ± 2.2	ND	179.2 ± 5.3	ND	151.4 ± 4.5
**FB**	0.6	5	-101.5 ± 3.8	-100.5 ± 2.4	0	0	43.0 ± 3.7	49.9 ± 4.3	36.3 ± 3.1	42.2 ± 3.7
	1		-150 ± 18.0	-164.6 ± 2.2	25.9 ± 1.2	0	62.4 ± 4.1	76.6 ± 2.8	52.7 ± 3.4	64.7 ± 2.3
	1.9		ND	-313.5 ± 5.3	ND	44.4 ± 5.1	ND	139.0 ± 9.4	ND	117.4 ± 7.9

^[a]^Rates for the whole conversion process. ^[b]^Linear region after 30 min. ^[c]^Extracellular 3HPA accumulation rate.

The specific glycerol consumption rate and 3HPA, 3HP and 1,3PDO production rates using wild-type (WT) and RPRB3007 mutant strain (M) of *L. reuteri* in batch (B) and variable-volume fed-batch (FB) modes of operation. Values with standard deviations represent the average of two independent replicates.

### Flux analysis and flux distribution through oxidative and reductive pathways in *L. reuteri* resting cells

**Figure 3:**
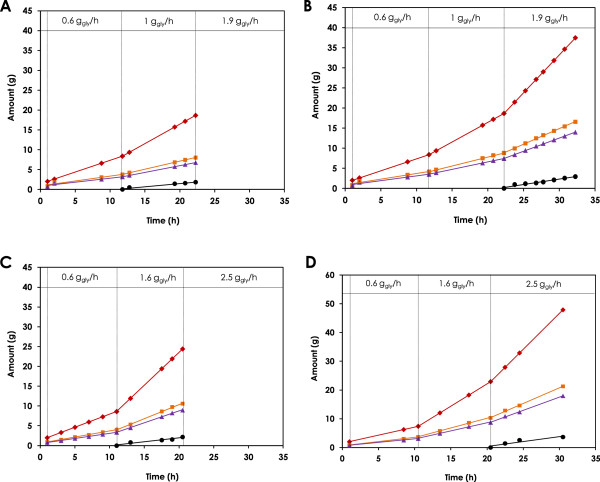
**Multi-step variable-volume fed-batch biotransformation of glycerol using resting cells of *L. reuteri.*** Multi-step variable-volume fed-batch biotransformation of glycerol using resting cells of wild-type **(A & C)** and RPRB3007 **(B & D)** strains of *L. reuteri* (6 g_CDW_/L). Experiments were conducted at pH 5 **(A & B)** and pH 7 **(C & D)**. After 1 h of batch biotransformation, glycerol (50 g/L) was fed at a rate of 0.6 g_gly_/h, 1.0 g_gly_/h and 1.9 g_gly_/h, respectively, for determination of the maximum flux through the reductive and oxidative branches of the Pdu pathway at pH 5. The corresponding feeding rates were 0.6 g_gly_/h, 1.6 g_gly_/h and 2.5 g_gly_/h, respectively, at pH 7. Consumed glycerol (◆), produced 3HP (■), 3HPA (●) and 1,3PDO (▲) are presented.

Bioconversion of glycerol to 1,3PDO and 3HP by *L. reuteri* is subject to different inhibitory mechanisms, which need to be avoided to achieve maximum yield and productivity. 3HPA besides being toxic to the cells is also inhibitory to the PduP enzyme at a concentration exceeding 0.6 g/L [34]. Moreover, high glycerol and 1,3PDO concentrations inhibit glycerol dehydratase activity [49]. A controlled variable-volume fed-batch operation with glycerol as a limiting substrate was designed, in which the feed was applied at a rate that allowed consumption of the entire glycerol and formation of 3HP and 1,3PDO as end products. To determine the maximum flux around 3HPA towards 3HP and 1,3PDO, the glycerol feeding rate was increased stepwise to a level that maximized the fluxes *ν*_*2*_ and *ν*_*3*_ (Figure 1). At this point, accumulation of 3HPA occurred (flux *ν*_*4*_) at a concentration that was low enough not to cause toxic or inhibitory effects. Since the availability of co-factors produced during cell growth phase could result in apparent rates in the initial hours of fed-batch biotransformation that are higher than the actual rates, data from the first 10 hours of the bioconversion was not utilized in the calculation of the fluxes.When the experiment was conducted at pH 5, no accumulation of the intermediate aldehyde was observed in the wild-type strain during operation in batch (1 h) and fed-batch at 0.6 g/h glycerol (Figure 3A). Complete conversion of glycerol to 3HP and 1,3PDO as the main end products was observed. The link between the oxidative and reductive branches through the co-factor regeneration reaction created a rigid node around 3HPA. The flux split ratio around 3HPA (ɸ) was ~50 mol% from 3HPA to 3HP and 1,3PDO, respectively. Subsequently, increasing the feeding rate to 1 g/h resulted in the production of 5.98 g/L of 3HP, 5.05 g/L of 1,3PDO, and accumulation of extracellular 3HPA to 1.36 g/L after 10 h (Figure 3A). The corresponding production rates were 0.40, 0.34 and 0.15 g/h, respectively.

Using the RPRB3007 strain at pH 5 showed no accumulation of the intermediate 3HPA during fed-batch operation at 0.6 and 1 g/h glycerol feeding rates. Complete conversion of glycerol to 6.57 g/L 3HP and 5.55 g/L 1,3PDO was achieved. Further increase in the feeding rate to 1.9 g/h glycerol between 22.3 h and 32.3 h resulted in accumulation of 3HPA to 1.7 g/L at the end of the biotransformation period. The production rates during the last 10 h of biotransformation were 0.66 g/h and 0.78 g/h for 1,3PDO and 3HP, respectively (Figure 3B). The maximum specific production rates obtained with the wild-type and RPRB3007 strains were confirmed by independent experiments where the glycerol feeding rate was maintained at the maximum rate determined for each strain (data not shown), which also revealed that the maximum specific production rates for the products were 2.2 fold higher with the RPRB3007 strain (Table 1).

Increasing the pH of the biotransformation reaction to 7 resulted in an increase in the specific production rates of 3HP and 1,3PDO to 110.8 and 93.7 mg/g_CDW_.h for the wild-type strain, and 179.2 and 151.4 mg/g_CDW_.h for the RPRB3007 strain, respectively (Figure 3C & 3D and Table 1). This is in agreement with the reported optimum pH for the activity of PduP [33], and is also close to the reported optimum pH of 6.2 for PduQ [50]. In this case, the final titers of 3HP and 1,3PDO were 10.6 and 9.0 g/L, respectively.

### Implications for the application of *L. reuteri* for production of 3HPA, 3HP and 1,3PDO

The results described above show that the use of resting cells of *L. reuteri* in combination with a 3HPA scavenger under batch mode of operation seems to be a promising approach for 3HPA production. While the RPRB3007 mutant strain with the overexpressed *pdu* operon exhibited almost two-fold higher specific production rate of 3HPA than the wild-type strain, there is no change in the susceptibility to 3HPA inhibition and requires the presence of the scavenger for aldehyde production. Fed-batch and immobilized-cell configurations have been used with the wild-type strain for the production of 3HPA as a carbohydrazide complex with improved yields [29] and should also be tested with the RPRB3007 strain. Considering the relatively high cost of carbohydrazide, it would also be useful to test other more cost-effective scavengers, or to isolate mutants with higher tolerance to 3HPA.

In the experiments for the production of 1,3PDO and 3HP with resting cells of the wild-type strain, the controlled glycerol feeding strategy allowed their co-production at a higher productivity (0.56 g_1,3PDO_/L.h and 0.66 g_3HP_/L.h) compared to that obtained using a recombinant strain of *L. reuteri* lacking glycerol dehydrogenase activity under batch operation (0.06 g_1,3PDO_/L.h and 0.07 g_3HP_/L.h) [22]. The corresponding rates using the RPRB3007 strain were even higher, 0.91 g_1,3PDO_/L.h and 1.08 g_3HP_/L.h. So far, the highest volumetric productivities reported for 1,3PDO and 3HP were 7.6 g/L.h and 9 g/L.h, respectively, obtained using resting cells of recombinant *E. coli* overexpressing the *L. reuteri* genes encoding glycerol dehydratase, its reactivation factor, and 1,3-propanediol oxidoreductase, as well as an *E. coli* K-12 aldehyde dehydrogenase [22]. In contrast to *L. reuteri*, the absence of protein shells (metabolosomes) in *E. coli* minimizes the mass transfer limitation of the substrate, intermediates and co-factors, and 3HPA is converted to 3HP in a single-step reaction.

The measured fluxes for the different steps in the Pdu pathway indicate that the rate of glycerol dehydration catalysed by GDH is at least 10 times faster than the subsequent reduction or oxidation of 3HPA to 1,3PDO and 3HP, respectively (Table 1). Hence for targeting the co-production of 3HP and 1,3PDO, the glycerol feeding rate should be controlled to maintain the flux *v*_*1*_ ≤ (*v*_*2*_ + *v*_*3*_). When the glycerol feeding rate is ≥ *v*_*1*_, 3HPA is accumulated as the main end product. MFA further suggests that the oxidative and/or reductive pathways could be critical targets for further metabolic engineering towards enhanced production of 3HP and 1,3PDO. Further metabolic engineering of the RPRB3007 strain could help in reducing the product inhibition, and improving the volumetric productivity and yield. Since the Pdu pathway is a non-growth associated pathway, metabolic engineering for enhanced production of 3HPA, 3HP and 1,3PDO is possible without interference with microbial growth. There seems to be no need for knocking out the gene encoding for glycerol dehydrogenase as done by Yasuda *et al.*[22], since no DHA production was observed in our experiments.

**Figure 4:**
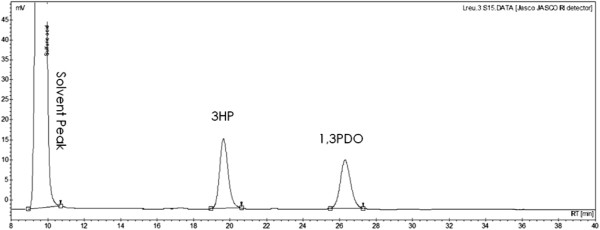
**Chromatographic profiles for 3HP and 1,3PDO production using resting cells of *L. reuteri.*** Chromatographic profile for the different products formed during transformation of glycerol to 3HP and 1,3PDO using cells of wild-type *L. reuteri* under resting conditions. The biotransformation conditions and the composition of the feeding solutions are described in the Methods section.

Despite the low volumetric productivity, there are many other factors that make the *L. reuteri*-based process desirable. The high purity of the resulting product mixture obtained using resting cells (Figure 4), greatly simplifies the downstream processing and minimizes production costs. In the process using recombinant strain of *K. pneumonia,* part of the glycerol is converted to dihydroxyacetone phosphate, which then enters the glycolytic pathway yielding lactic acid, acetic acid and other alcohols as the main by-products. In a study using recombinant *Pseudomonas denitrificans*, 3HP was further oxidized to malonate and utilized for cell growth resulting in decreased yield. Several by-products (lactic acid, succinic acid, acetic acid, ethanol and others) were indeed obtained as a result of glucose metabolism when the growing cells of *L. reuteri* were used (data not shown), which would lead to complicated downstream processing and increased costs even if the concentrations, volumetric- and specific production rates of 1,3PDO and 3HP were higher than those obtained using resting cells. The use of resting cells also makes process operation very simple, and moreover both the wild-type and RPRB3007 strains do not need to be grown in media with antibiotics that reduces operational costs.

Further studies to determine the extent of recyclability of the microbial biocatalyst as well as the maximum concentration of the two final products that can be tolerated are in progress.

## Conclusions

*L. reuteri* has great potential as a candidate for the industrial production of 3HPA, 3HP and 1,3PDO. The strain is amenable to metabolic engineering and a wide variety of methods for its genetic manipulation are available. Engineering of the *pdu* operon to increase the glycerol-utilization rate is a good strategy to increase specific production rates, and further manipulation could render a robust strain for industrial applications. This study presents a useful method for determination of metabolic fluxes of the Pdu pathway in *L. reuteri* with glycerol as substrate. The method not only provided a stepping stone for developing a production process for 3HPA or co-production of 3HP and 1,3PDO using whole resting cells of *L. reuteri* but also shed some light on important aspects to consider during process design to allow for cleaner production.

## Methods

### Materials

Glycerin Tech® (98%), a co-product of biodiesel production, and standard 3-hydroxypropionic acid (30% w/v) were provided by Perstorp AB, Sweden. Lactobacilli MRS broth (containing per liter: 10 g protease peptone, 10 g beef extract, 5 g yeast extract, 20 g dextrose, 1 g Tween 80, 2 g ammonium citrate, 5 g sodium acetate, 0.1 g magnesium sulfate, 0.05 g manganese sulfate and 2 g dipotassium phosphate) was a product of Difco (BD laboratories, Detroit, Michigan, USA). 1,3-Propanediol (99%) was obtained from Sigma-Aldrich (St Louis, MO, USA), glucose monohydrate from Prolabo (VWR International, Fontenay-sous-Bois, France), and 1,2-propanediol (1,2PDO) was from Merck (NJ, USA).

### Microorganisms and culture conditions

*L. reuteri* DSM 20016 and *L. reuteri* RPRB3007 with a modification in the catabolite repression element (CRE) upstream of the *pdu* operon [39], were used for the biotransformation of glycerol. Inocula were grown in 30-mL serum bottles containing 20 mL 55 g/L MRS and 20 mM 1,2-propanediol. The medium was added to the bottles, boiled, and bubbled with nitrogen gas. The bottles were then closed with rubber stoppers, and autoclaved at 121°C for 15 min. The sterilized medium was inoculated with 200 μL of a stock culture in 20% v/v glycerol and then incubated at 37°C for 16 h. Two hundred microliters of the resulting culture were transferred to 20 mL of fresh medium and incubated for 8 h under the same conditions. The resulting culture was used as inoculum in bioreactor studies.

### Production of the whole-cell biocatalyst for biotransformation of glycerol

*L. reuteri* cells were grown in a 3-L bioreactor (Applikon, Microbial Biobundle, The Netherlands). Monitoring and control of all the parameters was done through an *ez-control* unit. Stirrer speed was maintained at 200 rpm, temperature at 37°C and pH at 5.5 by addition of 5 N NH_4_OH. Anaerobic conditions were maintained through continuous bubbling of nitrogen gas. Twenty milliliters of the freshly prepared inoculum were aseptically added to 2-L fermentation medium containing 55 g/L MRS broth, 5 g/L 1,2-propanediol, and glucose at a final concentration of 40 g/L. Fermentation was conducted for 10 h after which the broth was collected and centrifuged at 15 000 × g and 4°C for 5 minutes. The supernatant was discarded and the cell pellet was used for the biotransformation of glycerol.

### Batch production of 3HPA from glycerol using resting cells of *L. reuteri*

Biotransformation of glycerol was done in a 1-L Biostat®-Q bioreactor (B. Braun Biotech International, Melsungen, Germany) with a 0.5-L working volume. The process was started by resuspending the *L. reuteri* cells obtained as described above, in 0.5 L solution containing 50 g/L glycerol and 50.6 g/L carbohydrazide to a final cell density of 6 g_CDW_/L. Glycerol biotransformation was performed at 37°C, pH 7, 500 rpm, with continuous nitrogen bubbling to maintain anaerobic conditions. Samples were collected and analyzed for glycerol, 3HP, 1,3PDO, and 3HPA, and the experiment was stopped when all the glycerol had been consumed.

The biotransformation kinetics were determined using the following equations:

–

$$\text{Volumetric}\;\text{production}\;\text{rate},Q_{p}\left(g/L.h\right)=\left[P_{\text{final}}–P_{\text{initial}}\right]/\Delta t$$

–

$$\text{Volumetric}\;\text{consumption}\;\text{rate},Q_{s}\left(g/L.h\right)=\left[S_{\text{final}}–S_{\text{initial}}\right]/\Delta t$$

–

$$\text{Specific}\;\text{production}\;\text{rate},q_{p}\left(\text{mg}/g_{\text{CDW}}.h\right)=Q_{p}\cdot 1000/X$$

–

$$\text{Specific}\;\text{consumption}\;\text{rate},q_{s}\left(\text{mg}/g_{\text{CDW}}.h\right)=Q_{s}\cdot 1000/X$$

where *P* and *S* are the concentrations of the products and substrate (g/L), respectively, *X* is the cell density (g_CDW_/L), and ∆*t* is the time elapsed between the initial and final conditions (h).

### Fed-batch production of 1,3PDO and 3HP from glycerol using resting cells of *L. reuteri*

Biotransformation of glycerol was done in a 3-L bioreactor (Applikon, The Netherlands) with a 1-L initial working volume. The process was started by resuspending the harvested *L. reuteri* cells from the biocatalyst-production step in a 1-L solution containing 2 g/L glycerol to a final density of 6 g_CDW_/L. After 1 h of batch biotransformation, fed-batch mode was started by feeding glycerol (50 g/L) at a rate of 12 mL/h (0.6 g_gly_/h) for 10 h. Subsequently, the feeding rate was increased to 31.1 mL/h (1.6 g_gly_/h) for 10 h, and finally to 50 mL/h (2.5 g_gly_/h) for 10 h. The biotransformation was performed at 37˚C, pH 7, 500 rpm, with continuous nitrogen bubbling to maintain anaerobic conditions. The pH was chosen based on the reported optimum for some of the enzymes of the Pdu pathway [34,50].

Since some studies for 1,3PDO production in *L. reuteri* have used acidic pH conditions, the experiment was also conducted at pH 5 [51]. In this case, the feeding rates and feeding periods were 12 mL/h (0.6 g_gly_/h) for 11 h, 19.8 mL/h (1 g_gly_/h) for 10 h, and 38.1 mL/h (1.9 g_gly_/h) for 10 h.

The feeding rates were determined according to a preliminary fed-batch experiment at a constant feeding rate. The feeding time (10 h) was chosen to ensure the stability of the measured fluxes before shifting to a higher feeding rate.

Samples were collected frequently and analyzed for glycerol, 3HP, 3HPA and 1,3PDO concentrations. The biotransformation kinetics were determined for each step (feeding rate) using the following equations:

–

$$\text{Production}\;\text{rate}\left(g/h\right)=\left[\left(P_{\text{final}}\cdot V_{\text{final}}\right)–\left(P_{\text{initial}}\cdot V_{\text{initial}}\right)\right]/\Delta t$$

–

$$\begin{array}{ll} \;\text{Consumption}\;\text{rate}\;\left(g/h\right) & =\left[\left(S_{\text{final}}\cdot V_{\text{final}}\right)-\left(\left(S_{\text{feed}}\cdot V_{\text{feed}}\right)\right)\right)\\ & \;\left(+\left(\left(S_{\text{initial}}\cdot V_{\text{initial}}\right)\right)\right]/\Delta t \end{array}$$

–

$$\text{Specific}\;\text{production}\;\text{rate},q_{P}\left(\text{mg}/g_{\text{CDW}}.h\right)=\text{production}\;\text{rate}\left(g/h\right)\cdot 1000/x$$

–

$$\text{Specific}\;\text{consumption}\;\text{rate},q_{S}\left(\text{mg}/g_{\text{CDW}}.h\right)=\text{consumption}\;\text{rate}\left(g/h\right)\cdot 1000/x$$

where *P* and *S* are the concentrations of the products and substrate (g/L), respectively, *V* is the reaction volume, *x* is the amount of the biocatalyst (g_CDW_), and ∆*t* is the time elapsed between the initial and final conditions (h).

### Analytical procedures

Cell growth was monitored by measuring optical density at 620 nm using a Ultrospec 1000 spectrophotometer (Pharmacia Biotech, Uppsala, Sweden) and then correlated with cell dry weight (CDW). For determination of the cell dry weight, 10 mL of the culture broth were centrifuged at 3893 × g for 20 minutes in a pre-dried (105°C for 2 h), pre-weighed 15 mL tube. The supernatant was removed and the cell pellet was dried at 105°C overnight and then weighed again. The difference in weight is equivalent to cell dry weight in 10 mL culture.

Glycerol, glucose, lactic acid, ethanol, acetic acid, 1,2-propanediol, propionaldehyde, propionic acid, 3HP, and 1,3PDO concentrations were determined by HPLC (JASCO, Tokyo, Japan) equipped with RI detector (ERC, Kawaguchi, Japan), a JASCO UV detector and a JASCO intelligent autosampler. Separation of the compounds was done on an Aminex HPX-87H chromatographic column connected to a guard column (Biorad, Richmond, CA, USA). The column temperature was maintained at 65°C in a chromatographic oven (Shimadzu, Tokyo, Japan). Samples from the bioreactor were diluted with Milli-*Q* quality water and mixed with 20% v/v sulfuric acid (20 μL/mL sample) and then filtered. A forty-microliter aliquot was injected in 0.5 mM H_2_SO_4_ mobile phase flowing at a rate of 0.4 mL/min. The retention times (min) for the different compounds were 13.890 (glucose), 18.317 (lactic acid), 19.500 (3HP), 20.208 (glycerol), 22.350 (acetic acid), 25.400 (1,2-propanediol), 25.876 (propionic acid), 26.400 (1,3PDO), 32.858 (ethanol), 33.290 (propionaldehyde) and 40.100 min (*n*-propanol).

For the determination of 3HPA concentration, a modified colorimetric method of Circle *et al.* (1945) [52] as described by Ulmer and Zeng (2007) [53] with acrolein as standard was used. Briefly, 1 mL of sample (diluted to be within the range of the assay) was mixed with 750 μL of 10 mM dl-tryptophan solution in 50 mM HCl and 3 mL of concentrated HCl (fuming 37%). The reaction mixture was incubated for 20 min at 37°C, and the resulting purple color was then measured spectrophotometrically at 560 nm.

### Statistical analysis

The represented kinetics are the average of two independent replicates ± standard deviation. The significance of the results was calculated using the Students’ T-test with P < 0.05 (95% significance).

## Competing interests

The authors declare that they have no competing interests.

## Authors’ contributions

TD and LPP designed and performed the laboratory experiments and data analysis as well as prepared the first draft of the manuscript. RAB provided the RPRB3007 strain. RHK, RAB and SHP contributed in the formulation of the idea and the revision of the manuscript. All authors read and approved the final manuscript.

## References

[B1] Jong E Higson A Walsh P Wellisch M Barbosa M Blaauw R Gosselink R Ree R Jorgensen H Mandl M McLaughlin M Smith MA Willke T Value Added Products from Biorefineries 2012 Report prepared on behalf of IEA Bioenergy, Task 42 Biorefinery

[B2] Werpy T Petersen G Aden A Bozell J Holladay J White J Manheim A Elliot D Lasure L Jones S Gerber M Ibsen K Lumberg L Kelley S Top Value Added Chemicals from Biomass, Volume 1—Results of Screening for Potential Candidates from Sugars and Synthesis Gas 2004 Oak Ridge, TN: U.S. Department of Energy available at http://www.eere.energy.gov/biomass/pdfs/35523.pdf

[B3] Vollenweider S Lacroix C 3-Hydroxypropionaldehyde: applications and perspectives of biotechnological production Appl Microbiol Biotechnol 2004 64 16 27 10.1007/s00253-003-1497-y 14669058

[B4] Kumar V Ashok S Park S Recent advances in biological production of 3-hydroxypropionic acid Biotechnol Adv 2013 31 945 961 10.1016/j.biotechadv.2013.02.008 23473969

[B5] Haas T Yu D Sauer J Arntz D Freund A Tacke T Process for the Production of 1,3-proapendiol by Hydrogenating 3-Hydroxypropionaldehyde 1998 WO 1998057913 A1

[B6] Powell JB Mullin SB Weider PR Eubanks DC Arhancet JP Process for Preparing 1,3-propanediol 1998 US Patent 5770776

[B7] Bauer R du Toit M Kossmann J Influence of environmental parameters on production of the acrolein precursor 3-hydroxypropionaldehyde by *Lactobacillus reuteri* DSMZ 20016 and its accumulation by wine lactobacilli Int J Food Microbiol 2010 137 28 31 10.1016/j.ijfoodmicro.2009.10.012 19897270

[B8] Ulmer C Deckwer WD Zeng AP Zweistufiger prozess zur herstellung von 1,3-propandiol und 3-hydroxypropionaldehyd aus glycerin Chem-Ing-Tech 2002 74 674 10.1002/1522-2640(200205)74:5<674::AID-CITE674>3.0.CO;2-O

[B9] Lüthi-Peng Q Scharer S Puhan Z Production and stability of 3-hydroxypropionaldehyde in *Lactobacillus reuteri* Appl Microbiol Biotechnol 2002 60 73 80 10.1007/s00253-002-1099-0 12382044

[B10] Della Pina C Falletta E Rossi M A green approach to chemical building blocks. The case of 3-hydroxypropanoic acid Green Chem 2011 13 1624 1632 10.1039/c1gc15052a

[B11] Lilga MA White JF Holladay JE Zacher AH Muzatko DS Orth RJ Method for Conversion of β-hydroxy Carbonyl Compounds 2010 US Patent 7687661

[B12] Banner T Fosmer A Jessen H Marasco E Rush B Veldhouse J De Souza M Microbial bioprocesses for industrial-scale chemical production Biocatalysis for Green Chemistry and Chemical Process Development 2011 John Wiley & Sons, Inc: Tao J and Kazlauskas R. Hoboken, NJ 429 467

[B13] Zeng A-P Biebl H Bulk chemicals from biotechnology: the case of 1,3-propanediol production and the new trends Advances in Biochemical Engineering/Biotechnology, Volume 74 2002 New York: Springer Berlin Heidelberg: Scheper T, Schügerl K, Zeng A-P 239 259 10.1007/3-540-45736-4_11 11991182

[B14] Steverding D Mikrobielle herstellung von 1,3-propandiol. fermentative biotechnologie Chem Unserer Zeit 2010 44 384 389 10.1002/ciuz.201000531

[B15] Werle P Morawietz M Lundmark S Sörensen K Karvinen E Lehtonen J Alcohols, polyhydric Ullmann's Encyclopedia of Industrial Chemistry, Volume 2 2000 Wiley-VCH Verlag GmbH & Co. KGaA: Weinheim 263 284

[B16] Nakamura CE Gatenby AA Hsu AK-h La Reau RD Haynie SL Diaz-torres M Trimbur DE Whited GM Nagarajan V Payne MS Picataggio SK Nair RV Method for the Production of 1,3-propanediol by Recombinant Microorganisms 2000 US Patent 6013494

[B17] BASF, Cargill and Novozymes Achieve Milestone in Bio-based Acrylic Acid Process [ http://www.basf.com/group/pressrelease/P-13-356]

[B18] Mirasol F Chemical profile: Biodiesel ICIS Chemical Business 2009

[B19] Statistics - EU biodiesel industry [ http://www.ebb-eu.org/stats.php]

[B20] Agarwal GP Glycerol Microbial Bioproducts. Edited by Fiechter A 1990 New York: Springer Berlin Heidelberg: [Scheper T, Belkin S, Doran PM, Endo I, Gu MB, Hu WS, Mattiasson B, Nielsen J, Stephanopoulos GN, Ulber R, Zeng A-P, Zhong J-J, Zhou W, Harald S (Series Editors): Advances in Biochemical Engineering/Biotechnology, vol 41.] 95 128

[B21] Wang ZX Zhuge J Fang HY Prior BA Glycerol production by microbial fermentation: A review Biotechnol Adv 2001 19 201 223 10.1016/S0734-9750(01)00060-X 14538083

[B22] Yasuda S Mukoyama M Horikawa H Toraya T Morita H Process for Producting 1,3-propanediol and or/3-hydroxypropionic Acid 2007 US Patent 20070148749 A1

[B23] Zhu JG Ji XJ Huang H Du J Li S Ding YY Production of 3-hydroxypropionic acid by recombinant *Klebsiella pneumoniae* based on aeration and ORP controlled strategy Korean J Chem Eng 2009 26 1679 1685 10.1007/s11814-009-0240-5

[B24] Ashok S Raj SM Rathnasingh C Park S Development of recombinant *Klebsiella pneumoniae ΔdhaT* strain for the co-production of 3-hydroxypropionic acid and 1,3-propanediol from glycerol Appl Microbiol Biot 2011 90 1253 1265 10.1007/s00253-011-3148-z 21336929

[B25] Huang Y Li Z Shimizu K Ye Q Simultaneous production of 3-hydroxypropionic acid and 1,3-propanediol from glycerol by a recombinant strain of *Klebsiella pneumoniae* Bioresour Technol 2012 103 351 359 10.1016/j.biortech.2011.10.022 22055092

[B26] Kumar V Sankaranarayanan M Durgapal M Zhou S Ko Y Ashok S Sarkar R Park S Simultaneous production of 3-hydroxypropionic acid and 1,3-propanediol from glycerol using resting cells of the lactate dehydrogenase-deficient recombinant *Klebsiella pneumoniae* overexpressing an aldehyde dehydrogenase Bioresour Technol 2013 135 555 563 23228456 10.1016/j.biortech.2012.11.018

[B27] Jiang XL Meng X Xian M Biosynthetic pathways for 3-hydroxypropionic acid production Appl Microbiol Biot 2009 82 995 1003 10.1007/s00253-009-1898-7 19221732

[B28] Sardari RRR Dishisha T Pyo SH Hatti-Kaul R Improved production of 3-hydroxypropionaldehyde by complex formation with bisulfite during biotransformation of glycerol Biotechnol Bioeng 2013 110 1243 1248 10.1002/bit.24787 23172314

[B29] Krauter H Willke T Vorlop K-D Production of high amounts of 3-hydroxypropionaldehyde from glycerol by *Lactobacillus reuteri* with strongly increased biocatalyst lifetime and productivity New Biotechnol 2012 29 211 217 10.1016/j.nbt.2011.06.015 21729774

[B30] Sardari RRR Dishisha T Pyo S-H Hatti-Kaul R Biotransformation of glycerol to 3-hydroxypropionaldehyde: Improved production by *in situ* complexation with bisulfite in a fed-batch mode and separation on anion exchanger J Biotechnol 2013 168 534 542 10.1016/j.jbiotec.2013.09.009 24060827

[B31] Talarico TL Casas IA Chung TC Dobrogosz WJ Production and isolation of reuterin, a growth inhibitor produced by *Lactobacillus reuteri* Antimicrob Agents Chemother 1988 32 1854 1858 10.1128/AAC.32.12.1854 3245697 PMC176032

[B32] Raj SM Rathnasingh C Jo JE Park S Production of 3-hydroxypropionic acid from glycerol by a novel recombinant *Escherichia coli* BL21 strain Process Biochem 2008 43 1440 1446 10.1016/j.procbio.2008.04.027

[B33] Sriramulu DD Liang M Hernandez-Romero D Raux-Deery E Lunsdorf H Parsons JB Warren MJ Prentice MB *Lactobacillus reuteri* DSM 20016 produces cobalamin-dependent diol dehydratase in metabolosomes and metabolizes 1,2-propanediol by disproportionation J Bacteriol 2008 190 4559 4567 10.1128/JB.01535-07 18469107 PMC2446795

[B34] Sabet-Azad R Linares-Pasten JA Torkelson L Sardari RRR Hatti-Kaul R Coenzyme A-acylating propionaldehyde dehydrogenase (PduP) from *Lactobacillus reuteri*: Kinetic characterization and molecular modeling Enzyme Microb Technol 2013 53 235 242 10.1016/j.enzmictec.2013.05.007 23931688

[B35] Stephanopoulos G Metabolic Fluxes and Metabolic Engineering Metab Eng 1999 1 1 11 10.1006/mben.1998.0101 10935750

[B36] Stephanopoulos GN Aristidou AA Nielsen J Metabolic Engineering: Principles and Methodologies 1998 San Diego, CA: Academic Press

[B37] van Gulik WM de Laat WTAM Vinke JL Heijnen JJ Application of metabolic flux analysis for the identification of metabolic bottlenecks in the biosynthesis of penicillin-G Biotech Bioeng 2000 68 602 618 10.1002/(SICI)1097-0290(20000620)68:6<602::AID-BIT3>3.0.CO;2-2 10799985

[B38] Kerfeld CA Heinhorst S Cannon GC Bacterial Microcompartments Annu Rev Microbiol 2010 64 391 408 10.1146/annurev.micro.112408.134211 20825353

[B39] van Pijkeren J-P Neoh KM Sirias D Findley AS Britton RA Exploring optimization parameters to increase ssDNA recombineering in *Lactococcus lactis* and *Lactobacillus reuteri* Bioengineered 2012 3 209 217 10.4161/bioe.21049 22750793 PMC3476877

[B40] Stevens MJA Vollenweider S Meile L Lacroix C 1,3-Propanediol dehydrogenases in *Lactobacillus reuteri*: impact on central metabolism and 3-hydroxypropionaldehyde production Microb Cell Fact 2011 10 61 69 10.1186/1475-2859-10-61 21812997 PMC3180264

[B41] Lüthi-Peng Q Dileme FB Puhan Z Effect of glucose on glycerol bioconversion by *Lactobacillus reuteri* Appl Microbiol Biot 2002 59 289 296 10.1007/s00253-002-1002-z 12111160

[B42] Luo LH Seo JW Baek JO Oh BR Heo SY Hong WK Kim DH Kim CH Identification and characterization of the propanediol utilization protein PduP of *Lactobacillus reuteri* for 3-hydroxypropionic acid production from glycerol Appl Microbiol Biot 2011 89 697 703 10.1007/s00253-010-2887-6 20890600

[B43] van Maris AJA Konings WN van Dijken JP Pronk JT Microbial export of lactic and 3-hydroxypropanoic acid: implications for industrial fermentation processes Metab Eng 2004 6 245 255 10.1016/j.ymben.2004.05.001 15491854

[B44] Toraya T Radical catalysis in coenzyme B12-dependent isomerization (eliminating) reactions Chem Rev 2003 103 2095 2127 10.1021/cr020428b 12797825

[B45] Arskold E Lohmeler-Vogel E Cao R Roos S Radstrom P van Niel EWJ Phosphoketolase pathway dominates in *Lactobacillus reuteri* ATCC 55730 containing dual pathways for glycolysis J Bacteriol 2008 190 206 212 10.1128/JB.01227-07 17965151 PMC2223725

[B46] Morita H Toh H Fukuda S Horikawa H Oshima K Suzuki T Murakami M Hisamatsu S Kato Y Takizawa T Fukuoka H Yoshimura T Itoh K O'Sullivan DJ McKay LL Ohno H Kikuchi J Masaoka T Hattori M Comparative genome analysis of *Lactobacillus reuteri* and *Lactobacillus fermentum* reveal a genomic island for reuterin and cobalamin production DNA Res 2008 15 151 161 10.1093/dnares/dsn009 18487258 PMC2650639

[B47] Cie A Lantz S Schlarp R Tzakas M Senior design reports (CBE): Renewable acrylic acid 2012 Working paper. University of Pennsylvania Available at: http://repository.upenn.edu/cbe_sdr/37/

[B48] Chen P Andersson DI Roth JR The control region of the *pdu/cob* regulon in *Salmonella typhimurium* J Bacteriol 1994 176 5474 5482 8071226 10.1128/jb.176.17.5474-5482.1994 PMC196736

[B49] Doleyres Y Beck P Vollenweider S Lacroix C Production of 3-hydroxypropionaldehyde using a two-step process with *Lactobacillus reuteri* Appl Microbiol Biot 2005 68 467 474 10.1007/s00253-005-1895-4 15682289

[B50] Talarico TL Axelsson LT Novotny J Fiuzat M Dobrogosz WJ Utilization of glycerol as a hydrogen acceptor by *Lactobacillus reuteri*: purification of 1,3-propanediol:NAD oxidoreductase Appl Environ Microb 1990 56 943 948 10.1128/aem.56.4.943-948.1990 PMC184326 16348177

[B51] El-Ziney MG Arneborg N Uyttendaele M Debevere J Jakobsen M Characterization of growth and metabolite production of *Lactobacillus reuteri* during glucose/glycerol cofermentation in batch and continuous cultures Biotechnol Lett 1998 20 913 916 10.1023/A:1005434316757

[B52] Circle SJ Stone L Boruff CS Acrolein determination by means of tryptophane: a colorimetric micromethod Ind Eng Chem 1945 17 259 262

[B53] Ulmer C Zeng AP Microbial production of 3-hydroxypropionaldehyde from glycerol bioconversion Chem Biochem Eng Q 2007 21 321 326

